# Relationship between anthropometric and biochemical changes of metabolic syndrome with retinal nerve fiber layer and macular thickness

**DOI:** 10.1371/journal.pone.0246830

**Published:** 2021-02-25

**Authors:** Sze Hui New, Sue Ngein Leow, Suresh Kumar Vasudevan, Idayu Badilla Idris, Seng Fai Tang, Norshamsiah Md Din

**Affiliations:** 1 Faculty of Medicine, Department of Ophthalmology, UKM Medical Center, Cheras, Kuala Lumpur, Malaysia; 2 Department of Ophthalmology, Hospital Sultanah Aminah Johor Bahru, Ministry of Health Malaysia, Johor, Malaysia; 3 Faculty of Medicine, Department of Community Health, UKM Medical Center, Cheras, Kuala Lumpur, Malaysia; University of Melbourne, AUSTRALIA

## Abstract

**Objective:**

To evaluate the retinal nerve fiber layer (RNFL) and macular thicknesses and identify systemic risk factors for thinning of these layers in patients with metabolic syndrome (MetS).

**Methodology:**

A cross-sectional observational study was performed on patients diagnosed with MetS and compared to normal controls. All patients underwent ophthalmic and anthropometric examination, serological and biochemical blood investigations; and ocular imaging using spectral-domain optical coherence tomography. Patients with ocular pathology were excluded. Unpaired t-test was used to compare mean thickness between the two groups. One-way ANOVA with Bonferroni correction for multiple comparisons was used to compare mean thickness between different tertiles of MetS parameters, and a generalized estimating equation was used to correct for inter-eye correlation and to assess association between mean thickness and covariates.

**Results:**

Two hundred and forty-eight eyes from 124 participants (1:1 ratio of MetS patients to controls) were included. Age ranged between 30 to 50 years old, and mean age was 40 ± 6.6 years. RNFL thickness was lower globally (93.6 ± 9.9 μm vs 99.0 ± 9.3, p<0.001) and in the inferior (124.5 ± 17.5 μm vs 131.0 ± 16.4 μm, p = 0.002), superior (117.2 ± 16.0 μm vs 126.3 ± 14.4 μm, p<0.001) and temporal (65.5 ± 10.2 μm vs 69.5 ± 9.8, p = 0.002) sectors in MetS patients compared to controls. Only the central (237.0 ± 14.0 μm vs 243.6 ± 18.0 μm, p = 0.002) and inferior parafoveal (307.8 ± 20.9 vs 314.6 ± 14.6, p = 0.004) area of the macula was significantly thinner. The inferior RNFL sector had the most difference (mean difference = 9.1 μm). The Generalized Estimating Equation found that, after adjusting for age, diastolic blood pressure, BMI, HDL and obesity; the number of MetS components and elevated triglyceride levels were independent risk factors for reduced thickness in global RNFL (β = -4.4, 95% CI = -7.29 to -1.5, p = 0.003) and inferior parafovea (β = -6.85, 95% CI = -11.58 to -2.13, p = 0.004) thickness respectively.

**Conclusion:**

RNFL thinning was seen more than macula thinning in MetS patients, suggesting RNFL susceptibility to neurodegeneration than the macula. A higher number of metabolic components and elevated triglyceride levels were independent risk factors for retinal thinning in this group of patients.

## Introduction

Metabolic syndrome (MetS) is a complex disturbance of an individual’s metabolism. Each component of MetS occurs either simultaneously or consecutively [1]. Both genetic and environmental factors have been implicated. It includes disturbances in lipid metabolism causing obesity and dyslipidemia; carbohydrate metabolism resulting in glucose intolerance and diabetes mellitus (DM); and hemodynamic disturbances resulting in arterial hypertension [2,3]. It is thought to be one of the most important medical problems of the 21^st^ century due to high-stress levels and sedentary lifestyles; coupled with hyperglucidic and hyper lipid food.

The global prevalence is estimated to be 20–25% [4]. The prevalence is higher (31.7%) among the Malaysian population, with females and individuals above 50 years old to be more significantly affected [5]. Prevalence by ethnicity follow the racial distribution in Malaysia with 57.0% Malay, 28.5% Chinese, 8.9% Indian, and 5.0% Indigenous Sarawakians affected [6]. The escalating prevalence of this syndrome imposes increased health burden and implications.

The retinal nerve fiber layer (RNFL) is the innermost retinal neural layer consisting of the unmyelinated axons of the retinal ganglion cells, making it ideal for assessment of central nervous system health and possible neurodegeneration [7]. The macula is the central 5 mm of the posterior pole, consisting of the fovea, a 1 mm-in-diameter area centered around a central depression called the foveola, specialized for high spatial acuity and color vision; the parafoveal area, a 2 mm wide ring surrounding the fovea, where the ganglion cell layer, inner nuclear layer, and outer plexiform layer are thickest; and the perifoveal zone, a ring approximately 2 mm wide next to the parafoveal area [8]. Optical coherence tomography (OCT) offers a non-contact and non-invasive imaging tool to measure these retinal layers by measuring the delay in the reflection of laser light from the retina.

Reduced RNFL and macular thickness have been shown to occur in many metabolic diseases such as obesity, diabetes mellitus, and hypertension. Karaca et al found reduced thickness in the inner layers of various areas of the macula in 29 eyes of MetS patients compared to controls [7]. Laiginhas et al found reduced RNFL thickness in obese patients going for bariatric surgery [9]. A systematic review by De Clerck et al evaluated the effect of diabetes alone on the retina and optic nerve head and found retinal degenerative changes were more evident when diabetic retinopathy and polyneuropathy were present [10]. Although both metabolic health status and obesity were significant risk factors for Primary Open Angle Glaucoma (POAG); metabolic status has been shown to be the more important risk factor [11]. Due to variability in results from these studies on different components of MetS, we aim to evaluate the RNFL and macular thicknesses together, investigate their associations with all MetS components and identify any independent risk factors for reduced thickness in both retinal areas.

## Methods

### Study population

This is a cross-sectional observational study conducted from June 2016 to October 2017 involving patients who attended the diabetic retinopathy screening clinic in Sultanah Aminah Hospital, Johor Bahru, Malaysia.

Eligible patients were those diagnosed with MetS according to the International Diabetes Federation (IDF) criteria 2006 [12], and aged between 30–50 years old. The control group was age-matched volunteers who had no known medical or ocular illnesses with body mass index (BMI) within normal limits of their respective standard deviation scores.

Individuals were excluded if they had elevated IOP > 22 mmHg, glaucoma, diabetic retinopathy, hypertensive retinopathy, high refractive errors within the range of -6.00 to +6.00, history of refractive surgery or laser therapy; and having any other ocular co-morbidities such as uveitis, retinal vascular occlusion, optic neuropathy, or retinitis pigmentosa. Patients with ocular trauma, opaque ocular media, or other systemic illnesses such as chronic renal failure, cerebrovascular accident, and brain tumor were also excluded.

The study was approved by the Medical Research and Ethics Committee (MREC) Malaysia and Malaysia National University (UKM) Research Ethics Committee (approval number GGPM-2016-080) in accordance with the tenets of the Declaration of Helsinki. Written informed consent was obtained from all participants.

### Metabolic syndrome

Metabolic syndrome (MetS) was defined following the New International Diabetes Federation definition 2006 [12] with the following criteria: presence of central obesity with waist circumference measuring >90 cm in men and >80 cm in women, plus any two of the following four factors: (i) raised triglyceride level: ≥150 mg/dL (1.69 mmol/L), or receiving specific triglyceride treatment; (ii) reduced HDL cholesterol: ≤40 mg/dL in men (1.03 mmol/L) or ≤50 mg/dL (1.29 mmol/L) in women, or receiving specific HDL treatment; (iii) high blood pressure (BP): systolic BP≥ 130 mmHg, or diastolic BP ≥ 85 mmHg, or treatment for previously diagnosed hypertension; and (iv) raised fasting plasma glucose: ≥100 mg/dL (5.6 mmol/L), or previously diagnosed type 2 diabetes. If the BMI is greater than 30 kg/m^2^, central obesity can be assured and waist circumference does not need to be measured.

### Anthropometric and biochemical measurements

Anthropometric measurements were performed on participants in light clothing and without shoes. Height was measured using a rigid Leicester stadiometer and weight with a digital weighing scale (model number: DEB 9322, Malaysia). BMI was calculated according to the Quetelet index which was formulated as weight/height^2^ in kilograms per meter square. Seated BP was measured with a sphygmomanometer. Three separate readings were measured at one-minute intervals and the average was taken for analysis. Waist circumference was measured at 2 cm above the umbilicus at the end of normal expiration.

Venous blood samples were collected after 6 hours of fasting. Blood was tested for fasting blood sugar (FBS), fasting lipid profile which included total cholesterol, triglycerides, low-density lipoprotein (LDL) and high-density lipoprotein (HDL) cholesterol; glycosylated hemoglobin (HbA1c), and renal profile.

### Ophthalmologic examination and OCT measurement

Ophthalmologic examinations were performed between 8:00 am and 11:00 am by the same examiner. This included visual acuity, refraction, IOP by using the Goldman applanation tonometry, central corneal thickness, gonioscopy by using Zeiss gonioscopy lens, slit lamp fundus examination after dilation with Phenylephrine and Tropicamide; and fundus color photography using a Topcon fundus camera (Topcon TRC 50-EX). Axial length was measured with IOL Master 500 (Carl Zeiss Meditec).

The peripapillary RNFL thickness was measured with Cirrus HD–OCT 5000 (Carl Zeiss Meditec), a spectral-domain OCT using circular scans located 3.4 mm from the center of the optic disc and recorded as global and four regional sectors (superior, inferior, nasal and temporal). The instrument’s 840-nm wavelength laser beam also generate a cube of data measuring 6×6 mm after scanning a series of 200 B-scans with 200 A-scans per B-scan which consists of 40,000 points in total. The optic nerve head algorithm was designed to precisely measure the neuro-retinal rim and give values for disc area and vertical cup-to-disc ratio.

Macular thickness measurements were taken from the inner limiting membrane to the midpoint of the retinal pigment epithelium. The macular cube 512×128 scan protocol performs 128 B-scans of 512 A-scans in a 6×6 mm scanning area. Macular thickness measurements were displayed as the mean of the following nine regions, as defined by the Early Treatment Diabetic Retinopathy Study (ETDRS): the central fovea, or the central macular thickness (CMT), defined as the average thickness in the central 1000 μm diameter; and the four inner and four outer quadrant subfields within 1–3 mm (parafovea) and 3–6 mm (perifovea) of the center respectively. The four subdivisions of the parafoveal and perifoveal areas were the superior, inferior, nasal, and temporal sectors.

Three images were taken from each participant, and the image with the highest quality score was chosen for analysis. All analyzed images had satisfactory (>5) quality score. Both eyes of each participant were included if they fulfill the inclusion and exclusion criteria.

### Statistical analysis

All collected data were analyzed using STATA, version 14 (Stata Corp). Normality of data was determined using the normal quantile plot. All categorical variables were compared using chi-square test and an unpaired *t-*test was used to compare means of retinal thickness between the two groups. A one-way ANOVA with Bonferroni correction for multiple comparisons were used to compare means between different tertiles of the MetS components.

Generalized Estimating Equations (GEE) adjusting for inter-eye correlation was used to model the difference in retinal thickness between different components of MetS. A p value of less than 0.004 to account for the 13 areas of the retina measured was considered statistically significant when one-way ANOVA and GEE were used. For all other tests, a p value of <0.05 was considered statistically significant.

## Results

### Demographic data

In total, 124 eyes of 62 MetS patients and 124 eyes of 62 healthy controls were examined, of whom 47% were Malays, 31% Chinese, and 22% Indian. The age ranged from 30 to 50 years old, and the mean age of all participants was 40 ± 6.6 years, with MetS patients being slightly older than controls (41.1 ± 7.0 vs 38.3 ± 6.0, p = 0.02). There was a slight female predilection with a male to female ratio of 1:1.48. Table 1 summarizes the baseline characteristics of the study’s participants. The vertical cup-to-disc ratio (VCDR) was slightly higher in MetS (0.55 ± 0.11) than the control group (0.51 ± 0.14) with borderline significance (p = 0.05) and perhaps not clinically significant. There was also no significant difference in IOP between the two groups (p = 0.62).

**Table 1:** Demographic data and clinical characteristics of participants.

Variables	Metabolic syndrome (n = 62)	Control (n = 62)	P-value
Age, mean ± SD, (years)	41.1 ± 7.0	38.3 ± 5.2	0.02*
Ethnicity, n (%)			0.078^#^
Malay	26 (42%)	31 (57.1%)	
Chinese	19 (30.6%)	21 (33.3%)	
Indian	17 (27.4%)	10 (9.5%)	
Male, n (%)	25 (40.3%)	25 (35.7%)	0.685^#^
BMI (kg/m^2^)	36.30 ± 6.83	22.71 ± 3.0	<0.001*
Systolic BP (mmHg)	141.4 ± 18.0	122.4 ± 8.0	<0.001*
Diastolic BP (mmHg)	82.0 ± 8.6	78.2 ± 2.6	0.001*
FBS (mmol/l)	7.1±2.5	4.7 ± 2.1	<0.001*
HbA1c (%)	7.64± 2.4	5.55 ± 0.38	<0.001*
HDL (mmol/l)	1.17 ±0.3	1.29±0.3	0.017*
Triglyceride (mmol/l)	1.83 ± 0.85	1.87 ± 0.22	0.77*
IOP (mmHg)	14.81 ± 1.91	14.96 ± 1.75	0.62*
Disc Area (mm)	2.17 ± 0.43	2.07 ± 0.35	0.162*
VCDR	0.55 ± 0.11	0.51 ± 0.14	0.05*
Axial length (mm)	24.34 ±0.46	24.36 ± 0.53	0.84*

* = unpaired t-test,

^#^ = chi square,

BMI = body mass index, IOP = intraocular pressure, VCDR = vertical cup-to disc ratio, FBS = fasting blood sugar, HbA1c = glycated hemoglobin, HDL = high density lipoprotein.

### RNFL and macular thickness between patients with metabolic syndrome and controls

There was no difference in the image quality of MetS patients and healthy controls (image quality score: 6.35 ± 0.99 vs 6.55 ± 1.0, p = 0.29). Eleven and 6 eyes of patients with MetS and healthy controls respectively had to be excluded because of poor OCT image quality. Therefore only 113 eyes of MetS and 118 eyes of controls were included for analysis. Table 2 summarizes the RNFL and macular thickness between the two groups.

**Table 2:** Comparison of retinal nerve fiber layer and macular thickness measurements between metabolic syndrome patients and healthy controls.

OCT parameters	Metabolic Syndrome (n = 113)	Control (n = 118)	P-value *
RNFL (Global, μm)	93.6 ± 9.9	99.0 ± 9.3	**<0.001**
RNFL (Superior, μm)	117.2 ± 16.0	126.3 ± 14.4	**<0.001**
RNFL (Inferior, μm)	124.5 ± 17.5	131.0 ± 16.4	**0.002**
RNFL (Nasal, μm)	67.2 ± 10.3	67.7 ± 9.5	0.70
RNFL (Temporal, μm)	65.5 ± 10.2	69.5 ± 9.8	**0.002**
CMT (μm)	237.0 ±14.0	243.6 ± 18.0	**0.002**
Parafoveal (Global, μm)	311.5 ± 17.6	315.4 ± 14.0	0.062
Parafoveal (Superior, μm)	316.0 ± 19.2	319.8 ± 17.2	0.2
Parafoveal (Inferior, μm)	307.8 ± 20.9	314.6 ± 14.6	**0.004**
Parafoveal (Nasal, μm)	318.1 ± 16.3	320.3 ± 15.9	0.3
Parafoveal (Temporal, μm)	303.6 ± 17.1	306.0 ± 18.9	0.32
Perifoveal (Global, μm)	274.2 ± 28.5	279.3 ± 11.9	0.07
Perifoveal (Superior, μm)	278.5 ± 18.5	282.6 ± 13.7	0.051
Perifoveal (Inferior, μm)	270.1 ± 18.0	270.5 ± 13.0	0.84
Perifoveal (Nasal, μm)	298.1 ± 14.1	300.7 ± 17.1	0.20
Perifoveal (Temporal, μm)	260.1 ± 12.8	262.0 ± 12.4	0.27

Data presented as mean ± SD,

* = unpaired t-test,

RNFL = retinal nerve fiber layer, CMT = central macular thickness.

RNFL thickness in MetS patients were thinner globally (93.6 ± 9.9 μm vs 99.0 ± 9.3, p<0.001); and in the superior (117.2 ± 16.0 μm vs 126.3 ± 14.4 μm, p<0.001), inferior (124.5 ± 17.5 μm vs 131.0 ± 16.4 μm, p = 0.002), and temporal (65.5 ± 10.2 μm vs 69.5 ± 9.8 μm, p = 0.002) sectors compared to controls. The difference is most seen in the inferior sector, with a mean difference of 9.1 μm compared to other sectors.

The CMT was also significantly thinner in MetS patients than controls (237.0 μm ±14.0 vs 243.6 ± 18.0 μm, p = 0.002) although the rest of the macular sectors showed no significant difference in thickness except the inferior parafoveal sector (307.8 ± 20.9 μm vs 314.6 ± 14.6 μm, p = 0.04).

Table 3 shows comparison of RNFL thickness in various components of metabolic abnormalities presented in tertiles. Correction for multiple comparison was performed using the Bonferroni method by dividing the intended p value (0.05) with 13 to account for 13 areas of the retina being tested. Therefore, a p value of 0.004 was taken as significant.

**Table 3:** Comparison of RNFL and macular thicknesses among patients with metabolic syndrome at different tertile levels of metabolic components.

Variables	Global RNFL (um)	Superior RNFL (um)	Global Parafoveal (um)	Inferior Parafoveal (um)	Global Perifoveal(um)	Central macula (um)
HbA1c 1^st^ tertile (n = 51)	95.12 ± 8.45	117.80 ± 15.49	316.88 ± 13.71	315.57 ± 15.91	272.42 ± 39.50	236.27 ± 14.04
2^nd^ tertile (n = 29)	95.03 ± 9.34	119.80 ±16.24	308.50 ± 19.98	298.96 ± 24.27	279.27 ± 16.49	238.38 ± 14.23
3^rd^ tertile (n = 33)	93.03 ± 11.58	113.97 ±16.68	305.93 ± 18.72	303.61 ± 20.71	272.51 ± 12.12	236.76 ± 14.10
P value *	0.05	0.34	0.013	**0.0008**. ⸹ p = 0.001 ^¶^ p = 0.023	0.54	0.81
FBS 1^st^ tertile (n = 41)	92.68 ± 8.66	113.51 ±15.31	310.69 ± 17.43	305.90 ± 17.89	278.34 ± 13.60	234.29 ± 12.49
2^nd^ tertile (n = 40)	95.67 ± 9.17	120.72 ±15.34	317.36 ± 14.34	314.32 ± 21.72	271.43 ± 44.71	239.17 ± 14.13
3^rd^ tertile (n = 32)	92.22 ± 11.85	117.5 ±17.25	305.32 ± 19.50	302.12 ± 21.80	272.37 ± 12.48	237.59 ± 15.48
P value *	0.25	0.13	0.013	0.035	0.51	0.28
SBP 1^st^ tertile (n = 40)	93.7 ± 9.68	116.92 ± 15.84	312.92 ± 18.99	308.95 ± 21.77	277.08± 14.21	236.22 ± 15.49
2^nd^ tertile (n = 42)	91.98 ± 10.05	115.79 ± 15.80	307.30 ± 18.40	305.17 ± 19.98	266.06 ± 43.30	239.31 ± 12.05
3^rd^ tertile (n = 31)	95.71 ± 9.76	119.45 ± 16.90	315.5 ± 13.30	309.93 ± 21.19	281.5 ± 7.69	234.71 ± 14.43
P value *	0.28	0.62	0.11	0.57	0.05	0.35
DBP 1^st^ tertile (n = 45)	90.24 ± 10.26	110.89 ± 15.53	314.05 ± 20.64	308.73 ± 24.91	270.05 ± 43.68	238.27 ± 14.39
2^nd^ tertile (n = 35)	94.97 ± 8.15	120.17 ± 11.51	307.85 ± 15.73	305.77 ± 16.94	274.06 ± 10.47	239.51 ± 12.48
3^rd^ tertile (n = 33)	96.76 ± 9.85	122.64 ± 18.24	312 ± 15.46	308.72 ± 18.99	280.03 ± 7.4	232.45 ± 14.35
P value *	0.009	**0.002** ⸹ p = 0.025 ^¶^ p = 0.003	0.29	0.79	0.31	0.08
HDL 1^st^ tertile (n = 45)	94.84 ± 10.41	119.89 ± 16.50	309.59 ± 17.09	306.89 ± 19.7	273.9 ± 9.52	237.31 ±12.3
2^nd^ tertile (n = 40)	91.8 ± 9.27	114.0 ± 15.55	309.45 ± 18.10	302.2 ± 22.55	278.62 ± 15.12	236.75 ± 14.5
3^rd^ tertile (n = 28)	94.21 ± 9.76	117.43 ± 15.75	317.62 ± 16.70	317.32 ± 17.29	268.4 ± 53.29	236.68 ± 15.55
P value*	0.34	0.24	0.10	0.011	0.34	0.97
Triglycerides 1^st^ tertile (n = 37)	94.54 ± 11.02	116.73 ± 15.61	310.38 ± 16.56	309.84 ±17.38	275.62 ± 10.95	235.48 ± 12.90
2^nd^ tertile (n = 44)	92.36 ± 9.76	116.34 ± 17.62	312.24 ± 18.56	309.54 ±22.09	270.50 ± 42.58	239.52 ± 15.37
3^rd^ tertile (n = 32)	94.25 ± 8.65	118.91 ± 14.45	311.88 ± 17.77	303.09 ± 22.68	277.67 ± 16.05	235.12 ± 13.15
P-value*	0.56	0.77	0.88	0.32	0.52	0.29
Overweight (BMI<30)	92 ± 11.30	115.71 ± 16.22	316.56 ± 14.46	312.33 ± 21.22	364.08 ± 56.66	239.5 ± 11.68
Obese (BMI ≥30)	94.04 ± 9.46	117.59 ± 16.07	310.18 ± 18.14	306.59 ±20.73	276.94 ± 12.76	236.27 ± 14.55
P-value*	0.36	0.6	0.11	0.23	0.05	0.31
3 Components	96.71 ± 8.70	119.32 ± 16.6	315.25 ± 17.08	310.91 ± 20.63	274.39 ±039.02	235.68 ± 14.28
4 Components	91.0 ± 7.01	114.18 ± 11.11	312.63 ± 17.20	309.36 ± 20.09	276.70 ± 11.17	242.14 ± 13.10
5 Components	90.14 ± 12.44	116 ± 18.7	303.28 ± 16.62	300.34 ± 19.97	271.44 ± 11.68	234.41 ± 13.50
P-value*	**0.0032** ⸹ p = 0.031 ^¶^ p = 0.009	0.34	0.0099	0.771	0.78	0.007

* = one-way ANOVA. P<0.004 is considered statistically significant to correct for multiple comparison.

^⸹^ = Bonferroni correction for ANOVA between 1^st^ and 2^nd^ tertile.

^¶^ = Bonferroni correction for ANOVA between 2^nd^ and 3^rd^ tertile. RNFL = retinal nerve fiber layer. All values are presented as mean ± standard deviation. Obesity was not included in the table as there was no thickness difference in any of the retinal sectors with obesity.

The global RNFL thickness was significantly thinner in patients with more MetS components, p = 0.0032. Those with five components have thinner global RNFL (90.12 ±12.44 μm) compared to four (91.0 ± 7.01μm), and three components (96.71 ± 8.70 μm).

The superior RNFL was significantly thinner in patients with higher diastolic BP, p = 0.002. Those in the first tertile had thinner superior RNFL (110.89 ± 15.53 μm) compared to the second (120.17 ±11.51 μm), and third tertile (122.64 ±18.24 μm).

The inferior parafoveal thickness was significantly different with different HbA1c levels, p = 0.0008; being thicker in the first (315.57± 15.91μm), and third tertile (303.61 ± 20.71μm).

The rest of the retinal thicknesses were not significantly different with other MetS components.

### Factors affecting RNFL and macular thickness

We performed generalized estimating equations (GEE) to assess factors affecting the RNFL and macular thicknesses against age, HbA1c, systolic BP, diastolic BP, HDL, triglyceride, fasting blood sugar, BMI, obesity and the number of MetS components. Table 4 summarizes the GEE results. We included only variables with significant results to simplify the table.

**Table 4:** Generalised estimating equations of OCT parameters and metabolic syndrome variables.

OCT parameters	Variables: Regression coefficient (β), (95% Confidence interval), P value*						
	Diastolic BP (mmHg)	Triglyceride (mmol/l)	HDL (mmol/l)	MetS components	HbA1c (%)	FBS (mmol/l)	BMI (kg/m^2^)
Global RNFL	β = 0.40 (0.05 to 0.75) P = 0.023	β = -0.9 (-3.53 to 1.73) P = 0.50	β = 0.47 (-5.05 to 6.0) P = 0.87	β = -4.4 (-7.29 to -1.5) **P = 0.003**	β = -0.27 (-1.89 to 1.33) P = 0.74	β = -0.04 (-1.45 to 1.52) P = 0.96	β = 0.09 (-(0.25 to 0.43) p = 0.61
Inferior RNFL	β = 0.65 (0.27 to 1.17) P = 0.041	β = -1.43 (-6.16 to 3.30) P = 0.55	β = -2.49 (-12.43 to 7.45) P = 0.62	β = -5.96 (-11.16 to -0.77) P = 0.024	β = -0.86 (-3.74 to 2.03) P = 0.56	β = -0.02 (-2.69 to 2.64) P = 0.98	β = 0.08 (-0.53 to 0.69) p = 0.78
Superior RNFL	β = 0.67 (0.13 to 1.21) P = 0.016	β = -0.33 (-4.49 to 3.81) P = 0.87	β = 0.54 (-8.15 to 9.22) P = 0.90	β = -3.92 (-8.47 to 0.64) P = 0.09	β = -0.21 (-2.74 to 2.32) P = 0.87	β = 0.05 (-2.28 to 2.39) P = 0.9	β = 0.27 (-0.26 to 0.80) p = 0.32
Temporal RNFL	β = 0.05 (-0.31 to 0.41) P = 0.79	β = -2.5 (-5.24 to -0.23) P = 0.073	β = -2.10 (-7.83 to 3.63) P = 0.47	β = -3.53 (-6.53 to -0.53) P = 0.021	β = -0.65 (-2.32 to 1.02) P = 0.44	β = 0.69 (-0.85 to 2.23) P = 0.38	β = -0.46 (-7.38 to 5.75) P = 0.795
Global parafoveal	β = 0.27 (-0.29 to 0.83) P = 0.35	β = -0.78 (-5.06 to 3.50) P = 0.72	β = 10.36 (1.37 to 19.35) P = 0.024	β = -5.09 (-9.79 to -0.39) P = 0.034	β = -1.27 (-3.88 to 1.34) P = 0.34	β = 1.56 (-0.84 to 3.98) P = 0.20	β = 0.51 (-0.04 to 1.06) p = 0.07
Inferior parafoveal	β = 0.60 (-0.02 to 1.22) P = 0.06	β = -6.85 (-11.58 to -2.13) **P = 0.0036**	β = 4.19 (-5.71 to 14.09) P = 0.41	β = -4.36 (-9.55 to 0.82) P = 0.09	β = -1.79 (-4.67 to 1.09) P = 0.22	β = 1.30 (-1.36 to 3.96) P = 0.34	β = 0.75 (0.14 to 1.35) p = 0.02
Superior parafoveal	β = 0.64 (0.035 to 1.24) P = 0.038	β = -0.95 (-5.56 to 3.66) P = 0.69	β = 9.9 (-2.36 to 25.92) P = 0.10	β = -4.53 (-9.59 to 0.53) P = 0.044	β = -2.17 (-4.98 to 0.63) P = 0.13	β = 2.65 (-0.06 to 5.25) P = 0.04	β = 0.37 (-0.21 to 0/97) p = 0.21
Superior perifoveal	β = 0.47 (-0.13 to 1.06) P = 0.12	β = 1.35 (-3.18 to 5.89) P = 0.56	β = 5.42 (-4.12 to 14.95) P = 0.26	β = -6.86 (-11.84 to -1.88) P = 0.007	β = 0.82 (-1.94 to 3.59) P = 0.56	β = 0.21 (-2.35 to 2.77) P = 0.87	β = 0.04 (-0.54 to 0.63 p = 0.88
Temporal perifoveal	β = 0.15 (-0.30 to 0.59) P = 0.51	β = 3.67 (2.67 to 7.09) P = 0.035	β = 7.28 (0.12 to 14.43) P = 0.046	β = -3.17 (-6.91 to 0.58) P = 0.09	β = -0.09 (-2.17 to 1.98) P = 0.93	β = 0.55 (-1.37 to 2.47) P = 0.57	β = 0.45 (0.01 to 0.89 p = 0.043

Although greater number of MetS components was significantly associated with reduced thickness in the global, inferior and temporal RNFL; and the global and superior parafoveal and superior perifoveal areas; after correcting for multiple comparisons, only the global RNFL reached statistical significance (p = 0.003). With every increase in one MetS components, the global RNFL reduced by 4.4 um (β = -4.4, 95% CI = -7.29 to -1.5, p = 0.003).

BMI and diastolic BP on the other hand was associated with mild increase in thickness which was perhaps not clinically significant, affecting areas in the inferior parafoveal and temporal perifoveal sectors for BMI; and in the global, inferior and superior RNFL, and superior perifoveal sectors for diastolic BP. All regression coefficient values were less than 1 um. There was no significant association between systolic BP and any variables. However, when Bonferroni correction was used, none of the associations reached statistical significance.

Elevated triglyceride levels was associated with reduced thickness only in the inferior and temporal parafoveal area, while HDL was associated with thicker global parafoveal and temporal perifoveal layers. However, when Bonferroni correction was applied, only triglyceride levels was associated with reduced thickness in the inferior parafoveal area. After adjusting for all other variables, with every 1 mmol/l increase in triglyceride, the inferior parafoveal area reduces by 6.85 um (β = -6.85, 95% CI = -11.58 to -2.13, p = 0.004).

While HbA1C did not show any significant associations in any of the areas, FBS showed significant association with increased thickness in the superior parafoveal sector, but did not reach statistical significance when Bonferroni correction was applied, p = 0.04.

## Discussion

In this cross-sectional study, we found that reduced RNFL thickness is more evident than reduced macular thickness in MetS patients compared to controls. The RNFL is thinner in all except the nasal sector but the macular layers were only significantly thinner in the inferior parafoveal and the central macula, suggesting the RNFL is susceptible to neurodegenerative changes towards derangement in biochemical and anthropometric parameters compared to the macula. The highest difference is seen at the inferior RNFL sector, similar to the preferential loss of the inferior sector of the neuroretinal rim in early glaucoma [13].

We also found that the number of MetS components and diastolic BP were associated with changes in more areas of the retina compared to the lipid (triglyceride and HDL levels) and sugar profiles (HbA1c and FBS). More number of MetS components was associated with thinner RNFL in all except the superior and nasal RNFL; and superior macula; whereas diastolic BP was associated with changes in the superior and inferior RNFL, and superior perifovea. This is in contrast to the lipid and sugar profiles which were associated with only two sectors of the macular region. While BMI was also associated with reduced thickness in two macular sectors, obesity, the primary criteria for MetS diagnosis, was not associated with any changes in retinal thickness. However, after applying Bonferroni correction for multiple comparisons, the only significant variables were the number of MetS components and triglyceride level, both negatively associated with the global RNFL and inferior parafovea thickness respectively.

Zarei et al examined RNFL thickness in patients with MetS and found lower thickness in the superior sectors, the reduction being more significant in patients with higher number of metabolic abnormalities after adjusting for age, gender, and laterality of examined eyes [14]. They found elevated triglyceride levels and fasting plasma glucose to be independently associated with lower RNFL thickness in the nasal sector and fasting plasma glucose in the temporal sector, partly agreeing with our findings.

In general, diastolic BP and BMI was associated with a very slight increase in RNFL thickness in various areas. The small regression coefficients indicate that the increments seen were all less than 1 μm which is perhaps not clinically significant. Furthermore, after correcting for multiple comparisons, these associations did not reach statistical significance. We also found no association between systolic BP with any of the retinal layers. This is in agreement with Karaca et al who studied these parameters on macular thickness in 29 patients with MetS and found no significant correlation between both systolic and diastolic BPs with macular layers [7]. We did not find DBP to be inversely associated with any of the OCT parameters after adjusting for other covariates, contrary to postulations linking hypertension with glaucomatous optic nerve changes [15,16].

Dyslipidemia is one of the core components of MetS affecting lipid metabolism, resulting in derangement of serum lipid constituents. Lipoprotein lipase is a crucial enzyme in breaking down the triglyceride form of fat, which is carried from various organs into the bloodstream by lipoprotein [17]. The deficiency of this enzyme results in accumulation of visceral fat and central obesity, and was found to result in retinal neurodegeneration especially in the nasal RNFL [17]. We found derangement of triglyceride and HDL were both associated with changes in the macula. Of the lipid parameters, HDL was associated with a mild increase in thickness in the global parafoveal and temporal perifoveal areas, possibly indicating a protective effect of HDL against neurodegeneration. Triglyceride on the other hand was associated with significant reduction in the inferior parafovea, even after applying Bonferroni correction for multiple comparison, suggesting a contributing factor for neurodegeneration at the macula. Both of these lipid parameters had no association with changes in any of the RNFL sectors.

Obesity causes vascular endothelial dysfunction and autonomic dysfunction, both resulting in abnormal ocular perfusion, impaired vascular supply to the optic nerve head and subsequent glaucomatous changes [17,18]. Adiposity, obesity, and obesity-related inflammatory factors such as leptin and interleukin-6 were found to be associated with RNFL thinning in children affecting all except the temporal quadrant [19]. However, in our cohort of adult patients, we did not find any association between obesity on any of the retinal layers. Although obesity has been regarded as the primary component of MetS [2], we did not find obesity to be a significant risk factor for thinning of the retinal layer when all covariates were examined together, contrary to the findings of other authors [8]. Laiginhas et al observed reduced thickness in the nasal and temporal sectors of the RNFL in their cohort of obese patients, after adjusting for confounders like hypertension, dyslipidemia, and diabetes [9]. Özen et al also observed a general decrease in RNFL thickness in non-diabetic obese children and adolescents (10–18 years old), which were more reduced in the inferior quadrant, similar to our findings in our cohort of adult patients [20]. Jung et al in their population based prospective study reported that patients who are metabolically healthy and obese are at higher risk of developing POAG compared to metabolically healthy and non-obese patients, suggesting that obesity carries some risk for POAG [11].

Perhaps the most significant contributing factor to changes in the retinal thickness was the number of MetS components. We found persistent negative associations with all retinal layers, after adjusting for all other confounders. The significant associations were in most RNFL sectors and some macular sectors, indicating a cumulative effect of the MetS components on retinal neurodegeneration especially at the peripapillary RNFL area.

We found FBS to be associated with only one macular sector and no RNFL sectors, although this association did not reach statistical significance after correction. Although there was a significant difference in both FBS and HbA1c between the MetS patients and controls, the association of these two diabetic parameters and the retina was not evident in our patients’ eyes without diabetic retinopathy. A systematic review has suggested that retinal degenerative changes were only evident when more severe DR and polyneuropathy were present [10], although microscopically, accumulation of glycation end products were found in and around the optic nerve head [21]. This explains our results and the results of other studies that found no difference in macular thickness or macular volume between diabetic patients with no or mild DR and non-diabetic individuals [22]. There are, however, conflicting reports on the association between RNFL thickness and diabetic parameters with those who showed difference [23] and those who did not [24].

In any case, our findings suggest that decreased retinal thickness involving the RNFL and macula in patients with MetS may indicate early neurodegenerative changes, such as loss or degeneration of glial cells, making them susceptible to diseases such as glaucoma. We found individuals with MetS have thinner RNFL and slightly higher VCDR, both of which are also seen in glaucoma. Glaucoma causes thinning of the RNFL as early as 6 years before Humphrey visual field loss [22], and detecting loss of RNFL thickness may help in early diagnosis of glaucoma.

We acknowledge the following limitations in our study. There was a lack of data pertaining to the diagnosis of normal-tension glaucoma in our participants, which is a possible confounder in the assessment of the retinal layers [25,26]. Secondly, this was a cross-sectional study in a single center, and a causative link cannot be established between the MetS components and the OCT parameters. The effect of fluctuations in the metabolic abnormalities to the retinal layers is impossible to be assessed with this study design, and whether the changes in thickness also fluctuates alongside fluctuations in the biochemical and anthropometric parameters. Lastly, clinical measures of diabetic peripheral neuropathy were not evaluated and thus not factored into the statistical analysis [27].

## Conclusion

Reduced thickness was more prominent in the RNFL than the macula of MetS patients, suggesting the RNFL susceptibility over the macula to undergo neurodegeneration. These changes were largely attributed by deranged lipid parameters and higher number of MetS components. We recommend further studies to establish whether this susceptibility will manifest as glaucomatous disc damage and whether the observed reduction in RNFL thicknesses may be corrected if metabolic anomalies are stabilized.

## Supporting information

S1 Data (CSV)
